# Role of TAF4 in Transcriptional Activation by Rta of Epstein-Barr Virus

**DOI:** 10.1371/journal.pone.0054075

**Published:** 2013-01-10

**Authors:** Ya-Chun Yang, Li-Kwan Chang

**Affiliations:** Department of Biochemical Science and Technology, College of Life Science, National Taiwan University, Taipei, Taiwan; Ecole Normale Supérieure de Lyon, France

## Abstract

Epstein-Barr virus (EBV) expresses an immediate-early protein, Rta, to activate the transcription of EBV lytic genes. This protein usually binds to Rta-response elements or interacts with Sp1 or Zta via a mediator protein, MCAF1, to activate transcription. Rta is also known to interact with TBP and TFIIB to activate transcription. This study finds that Rta interacts with TAF4, a component of TFIID complex, *in vitro* and *in vivo*, and on the TATA sequence in the BcLF1 promoter. Rta also interacts with TAF4 and Sp1 on Sp1-binding sequences on TATA-less promoters, including those of BNLF1, BALF5, and the human androgen receptor. These interactions are important to the transcriptional activation of these genes by Rta since introducing TAF4 shRNA substantially reduces the ability of Rta to activate these promoters. This investigation reveals how Rta interacts with TFIID to stimulate transcription.

## Introduction

Although Epstein-Barr virus is normally maintained under latent conditions in B lymphocytes after infection, the virus must enter a lytic cycle to produce infectious virions. In the immediate-early stage of the lytic cycle, EBV expresses Rta and Zta, which are encoded by BRLF1 and BZLF1, respectively, to activate the transcription of lytic genes [1]–[5]. As is commonly known, Rta binds to a defined sequence of Rta-response elements (RRE) in EBV lytic promoters, including those of BALF5, BMLF1 and BLLF1, to activate transcription [4], [6]–[11]. However, Rta also activates transcription via a mechanism that is independent of RRE binding. For example, Rta interacts with Sp1 via an intermediary protein, MCAF1, to form a complex on Sp1-binding sites that autoregulates its own transcription and enhances the transcription that is regulated by Sp1 [12], [13]. Rta also activates the transcription of BALF5 by interacting with USF and E2F instead of binding to an RRE [10]. Moreover, Rta modulates the phosphorylation of the p38, JNK and ERK signaling pathways, causing the binding of phosphorylated ATF2 to the ZII element in the BZLF1 promoter to activate Zta expression [14], [15]. Rta also interacts with Oct-1 to enhance the transcription of BZLF1 [16] and with TSG101 to activate the expression of EBV late genes, including BcLF1, BDLF3, BILF2, BLLF1, and BLRF2 [17]. Our earlier study demonstrated that MCAF1 simultaneously interacts with Rta and Zta, allowing Rta and Zta to activate transcription synergistically [18]. Furthermore, Rta is conjugated with SUMO-1 via interaction with SUMO E2 and E3 ligases, including PIAS1, PIASxα, and PIASxβ [19], [20], increasing the transactivation activity of Rta. Additionally, LF2 regulates viral replication by binding to Rta and altering the subcellular localization of Rta [21].

Transcriptional activation in eukaryotes is a complex process that involves numerous transcriptional and chromatin-remodelling factors. The general transcription factor TFIID, which includes the TATA-binding protein (TBP) and 13 TBP-associated factors (TAFs), recognizes and binds to the TATA box. After the binding, a stepwise assembly of a transcriptional preinitiation complex (PIC) then follows by recruiting other general transcription factors, including TFIIA, TFIIB, TFIIE, TFIIF and TFIIH, and RNA polymerase II [22], [23]. TFIID is known to initiate the TATA-dependent transcription and also activates a promoter that lacks a TATA box by interacting with TAFs or other transcriptional activators, such as Sp1 [23]–[26].

Human TAF4 (TAFII130/135), a human homolog of *Drosophila* TAFII110, is considered a core subunit in TFIID due to its role in stabilizing the holo-TFIID complex [27]. TAF4 is a transcriptional coactivator that contains four glutamine-rich domains that interact with Sp1, CREB and huntingtin [28]–[30]. However, the preferential interaction of Sp1 with mutant huntingtin interferes with the functions of Sp1 and TAF4, resulting in the inhibition of binding of Sp1 to DNA, which is associated with the pathogenesis of Huntington's disease [30]. Additionally, TAF4 affects the transactivation activities of viral genes by interacting with a number of viral proteins. For instance, the overexpression of TAF4 stimulates the activation of transcription by the small T antigen to promote SV40 DNA replication [31]. TAF4 also interacts with human adenovirus E1A protein to inhibit transcription [32] and promotes the function of STAT2 in Type-I interferon (IFN)-stimulated transcription by forming a non-classical TBP-free acetyltransferase complex, which prevents the transcriptional shutoff of host genes during poliovirus infection [33]. TAF4 also activates the transcription from a TATA-less promoter that contains a downstream core promoter element (DPE) [27]. The TATA-less promoters are involved in the transcription of many cellular and viral genes, including those that code for dihydrofolate reductase (DHFR), c-H-ras, adenosine deaminase, TGF-β, thymidylatesynthetase, human androgen receptor (hAR), SV-40 late gene products, and LMP1 and DNA polymerase (BALF5) of EBV [10], [34]–[37]. These TATA-less promoters typically contain Sp1-binding sequences. Through the interaction between Sp1 and TAF4, other transcription factors and RNA polymerase II are recruited to initiate transcription from a promoter that lacks a TATA sequence [28], [35]. Rta is a potent transcriptional factor that activates many viral and cellular genes through interaction with both TBP and TFIIB [38]. This study demonstrates that Rta enhances transcription via the interaction with TAF4.

## Methods

### Cell lines and EBV lytic induction

P3HR1 (ATCC HTB-62), a Burkitt's lymphoma cell line that contains EBV [39], was cultured in RPMI 1640 medium containing 10% fetal calf serum. 293T cells (ATCC CRL-11268) were cultured in Dulbecco's modified Eagle's medium (DMEM) containing 10% fetal calf serum. P3HR1 cells were treated with 30 ng/ml 12-*O*-tetradecanoylphobol-13-acetate (TPA) and 3 mM sodium butyrate to activate the EBV lytic cycle [40], [41].

### Plasmids

Plasmid pGEX-4T1, which expresses GST, was purchased from Amersham Biosciences. Plasmid pBSK-TAF4, which contains a TAF4 gene, was provided by Naoko Tanese [29]. Plasmid pGEX-TAF4, which encodes GST-TAF4, was constructed by inserting a 3.9-kb *Eco*RI fragment, which contains the TAF4 gene, from pBSK-TAF4 into the *Eco*RI site in pGEX-4T3 (Amersham Biosciences) to generate pGEX-TAF4. Plasmid pEGFP-TAF4, which expresses TAF4 that is fused to GFP, was constructed by digesting pGEX-TAF4 with *Eco*RI and *Xho*I and then inserting the TAF4 DNA fragment into the *Eco*RI-*Sal*I sites in pEGFP-C1 (Clontech). Plasmid pEGFP-TAF4-NM, which encodes the region in TAF4 from amino acid 1 to 701, was constructed by digesting pEGFP-TAF4 with *Cla*I and *Xho*I, eluting the digested fragment and treating with the DNA polymerase I Klenow fragment, and then inserted into pEGFP-C1. Plasmid pEGFP-TAF4-C, which expresses the C-terminal 246-amino acid region in TAF4 that is fused to GFP, was constructed by inserting a PCR-amplified TAF4 DNA fragment into the *Eco*RI-*Sal*I sites in pEGFP-C1. Plasmid pEGFP-Rta was provided by T. Y. Hsu [42]. Plasmids pCMV-R, pET-Rta, pBMRF1-luc and pBMRF1-mRRE were described elsewhere [18], [19]. Plasmids that express deletion mutants of GFP-Rta, including GFP-N190, GFP-N191/415 and GFP-Rev, which encodes Rta regions from amino acids 1–190, 191–415 and 416–605, respectively, were constructed by inserting a PCR-amplified BRLF1 fragment into the *Eco*RI-*Xho*I sites in pEGFP-C1. Plasmids pTRL1-luc, pmTRL1-luc, pBALF5-luc and phAR-luc were constructed by inserting PCR fragments that had been amplified using primer sets TRL1-H3 (5′-CCCAAGCTTGAATGAGGTGGCGGATT)/TRL1-X (5′-CCGCTCGAGCCGCGGGGGAAGGCCAC), TRL1-H3 (5′-CCCAAGCTTGAATGAGGTGGCGGATT)/mTRL1-X (5′-CCGCTCGAGCCGCGGGGGAAGGACCAGCTGCCTCC), BALF5(+170) (5′-AGATGTCGCAGCAGCCG)/BALF5(+16)(5′-CCCAAGCTTCCACTGAGGGTCCGGCC), and hAR(+140) (5′-CCGCTCGAGTAGCGCGCGGTGAGGGGA)/hAR(+57) (5′-CCCAAGCTTCTCTGGGCTTGCTCCGGA), respectively, into pGL2-basic (Promega) at the *Hin*dIII-*Xho*I sites. Plasmid pmBALF5-luc contains the same sequences as pBALF5-luc, but with the Sp1-binding sequence mutated from 5′-CCGCCC to 5′-AAGCCC. Plasmid pmhAR-luc has the same sequences as is present in phAR-luc, but 5′-GGGGCGGG was changed to 5′-GTGGGTTG. Plasmids pGL2-F23 was constructed by inserting a 23-bp double-strand DNA fragment that contains the region from nucleotide -38 to -16 in the BcLF1 promoter [43] into pGL2-basic.

### GST pull-down assay

GST, GST-TAF4, and His-Rta were purified from *E. coli* BL21(DE3) using glutathione-Sepharose 4B beads (Amersham Biosciences) according to a method described earlier [19].

### Immunoprecipitation

P3HR1 cells were treated or untreated with sodium butyrate and TPA for 24 hr to induce lytic cycle. A lysate was prepared using mRIPA buffer (50 mM Tris-HCl, pH 7.8, 150 mM NaCl, 5 mM EDTA, 0.5% Triton X-100, 0.5% Nonidet P-40) [13]. Monoclonal mouse anti-Rta antibody (1∶500 dilution) (Argene), or mouse anti-TAF4 antibody (1∶1000) (BD Biosciences) was added to the supernatant and incubated at 4°C for 1 hr. The normal mouse IgG (Santa Cruz Biotechnology) was used as a negative control. Protein G agarose beads (30 µl) (Oncogene) were then added to the lysate. After shaking for 1 hr at 4°C, the beads were collected by centrifugation and washed three times with mRIPA buffer. Proteins binding to the beads were eluted by adding 20 µl 2X electrophoresis sample buffer and analyzed by immunoblotting using anti-Rta and anti-TAF4 antibodies.

### Immunofluorescence analysis

P3HR1 cells were transfected with pEGFP-C1 or pEGFP-TAF4 and then treated with TPA and sodium butyrate to activate the EBV lytic cycle. After culturing for 24 hr, cells were harvested by centrifugation, plated on poly-L-Lysine (Sigma)-coated coverslips, and fixed with 4% paraformaldehyde in phosphate-buffered saline (PBS) for 30 min. Immunostaining was performed using anti-Rta monoclonal antibody. Cells were then treated with rhodamine-conjugated goat anti-mouse IgG polyclonal antibody (DAKO). Nuclei were visualized by staining using 5 µg/ml of 4′-6-diamidino-2-phenylindole (DAPI). Finally, cells were examined under a Zeiss confocal laser-scanning microscope (Model LSM780).

### DNA affinity precipitation assay (DAPA)

P3HR1 cells that had been treated with TPA and sodium butyrate were lysed in mRIPA buffer. The lysate was mixed with 300 ng 5′-biotin end-labeled double-stranded probe, F23, mF23, TR-L1, mTR-L1, hAR or mhAR in a binding buffer that contained 60 mM KCl, 12 mM HEPES, pH 7.9, 4 mM Tris-HCl, 5% glycerol, 0.5 mM EDTA, 1 mM DTT and 10 µg/ml each of leupeptin, aprotinin, and 4-(2-aminoethyl)-benzenesulfonyl fluoride. After it had been incubated under rotation for 1 hr at 4°C, DNA-protein complexes were captured using 100 µl Streptavidin MagneSphere Paramagnetic particles (Promega), incubated for 1 hr, and then washed five times in the binding buffer. Finally, precipitated DNA-protein complexes were mixed in 2X electrophoresis sample buffer and proteins were detected by immunoblotting. Probe F23 contains the sequence that covers the region from -38 to -16 in the BcLF1 promoter (5′-GAATTATTAAACCGGGTGGCAGC). Probe mF23 includes the same sequence as probe F23 except that the 5′-TATTAAA were changed to 5′-TGTTGAA. TR-L1 contains the sequence between nucleotide 170121 and 170154 in the EBV genome (GenBank accession number V01555) (5′-CCGCGGGGGAAGGCCACGCCCCCTCCACTTTTTC). Probe mTR-L1, which contains the same sequence as in TR-L1 except for a mutated Sp1 site (5′- CCGCGGGGGAAGGACCAGCTGCCTCCACTTTTTC), was used as a negative control. Probe hAR contained the nucleotide sequence from -32 to -63 in the human androgen receptor promoter (5′-AGGAGGCCGGCCCGGTGGGGGCGGGACCCGAC) [44], and probe mhAR contains a similar sequence as hAR except for the Sp1 site was mutated (5′-AGGAGGCCGGCCCGGTGGGTTCGGGACCCGAC).

### Chromatin immunoprecipitation (ChIP) assay

ChIP assay was conducted with P3HR1 cells as described previously [45]. Briefly, P3HR1 cells (1×10^7^) were treated with TPA and sodium butyrate to induce the EBV lytic cycle or treated with DMSO as a control. Cells were mixed with 1% formaldehyde and incubated under shaking for 10 min to crosslink proteins and DNA. After sonication, crosslinked DNA-protein complexes were immunoprecipitated using anti-Sp1 (Santa Cruz Biotechnology), anti-TAF4, or anti-Rta antibody. The presence of specific DNA fragments in the precipitates was detected by qPCR as described elsewhere [18]. Standard curves were generated using serial dilutions of input DNA (1000, 100, 10, 1 and 0.1%). The Ct value of each reaction was quantified against the standard curve. Primers used for amplifying the TR-L1 promoter were 5′-AGAATGAGGTGGCGGATTCA and 5′-CGCCCCCTCCACTTTTTC; the BALF5, 5′-CGTGAGGGAAATAACCAGGATC and 5′-TGGTTTCATCTCCTTCTTCAAAAAC; the hAR promoter, 5′-GGAGGTGGGAAGGCAAGGA and 5′-TGGAGAGCAAATGCAACAGTTT; the BcLF1 promoter, 5′-GCAGAGGACCATATTTCGCAGTCTG and 5′-GCTGCCACCCGGTTTAATAATTC.

### Transient transfection assay

Plasmids were purified from *E. coli* using a plasmid purification kit (Qiagen). P3HR1 cells (5×10^6^) were mixed with 5 µg plasmids in 300 µl cultured medium and transfected by electroporation at 975 µF and 0.2 V using a BTX ECM630 electroporator (BTX Instrument). 293T cells were transfected using TurboFect (Fermentas). Luciferase assay was monitored using a luminometer (Orion II; Berthod) as described elsewhere [46]. Each transfection experiment was performed at least three times, and each sample in the experiment was prepared in duplicate.

### RNAi

The shRNA of TAF4 was purchased from the National RNAi Core Facility, Genomic Research Center, Academia Sinica. Two plasmids (300 ng) that expressed TAF4 shRNA (clone ID: TRCN0000000004 and TRCN0000000005) were transfected into 293T cells using TurboFect (Fermentas). Inhibition of TAF4 expression was examined at 48 hr after transfection.

## Results

### Interaction between Rta and TAF4 *in vitro*

**Figure 1:**
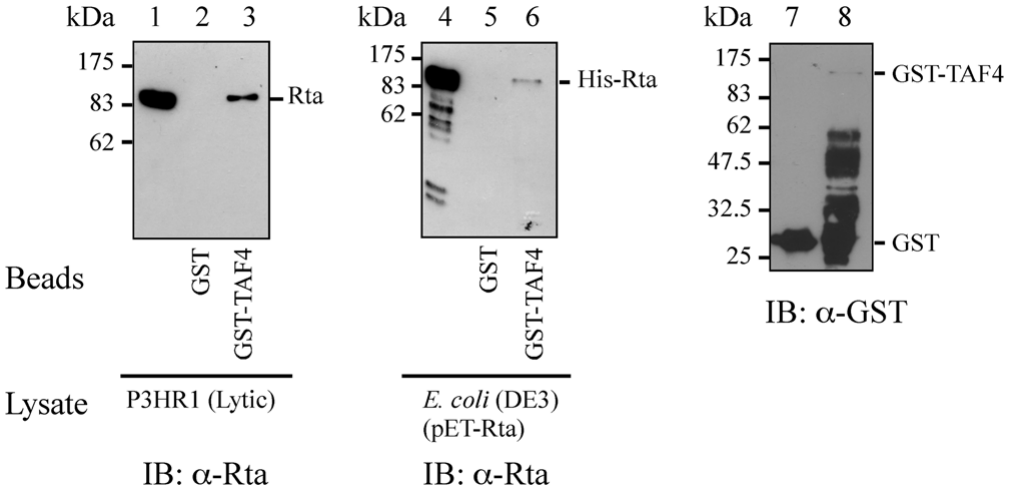
Interaction between Rta and TAF4 *in vitro*. GST-TAF4 (lanes 3 and 6) or GST (lanes 2, 5) was added to the lysate prepared from P3HR1 cells that had been treated with TPA and sodium butyrate (lanes 1–3) or from *E. coli* BL21(DE3)(pET-Rta) (lanes 4–6). Proteins bound to GST-TAF4 were pulled down by glutathione-Sepharose beads and analyzed by immunoblotting (IB) with anti-Rta antibody. Lanes 1 and 4 were loaded with 5% of cell lysate. GST or GST-TAF4 bound to glutathione-Sepharose beads were analyzed by immunoblotting (IB) with anti-GST antibody (lanes 7, 8).

Since both Rta and TAF4 interact with Sp1 [28], [30], [35], this study investigated whether Rta interacts with TAF4 to promote transcription. GST-pulldown assay was performed using bacterially expressed GST-TAF4 fusion protein (Fig. 1, lane 8) to investigate the interaction between Rta and TAF4. Assay results revealed that Rta expressed in the P3HR1 cells after lytic induction was retained by GST-TAF4 that was bound to glutathione-Sepharose beads but was not pulled down by GST-glutathione-Sepharose beads (Fig. 1, lanes 1–3). A similar assay was carried out by adding the *E. coli* BL21(DE3)(pET-Rta) lysate to GST- or GST-TAF4-glutathione-Sepharose beads. The results indicated that bacterially expressed His-Rta was retained by GST-TAF4-glutathione-Sepharose but not by GST-glutathione-Sepharose beads (Fig. 1, lanes 4–6), confirming the direct interaction between Rta and TAF4.

### Interaction between Rta and TAF4 *in vivo*

**Figure 2:**
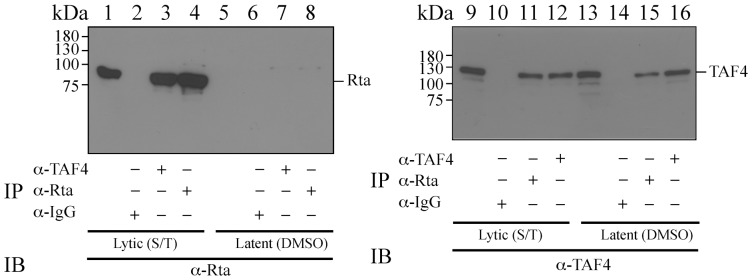
Coimmunoprecipitation of Rta and TAF4. Interaction between TAF4 and Rta was examined using P3HR1 cells that were treated with DMSO (lanes 5–8; 13–16) or TPA and sodium butyrate (lanes 1–4; 9–12) for 24 hr. Proteins in the cell lysate were immunoprecipitated with anti-Rta (lanes 4, 8, 11, 15), anti-TAF4 (lanes 3, 7, 12, 16) or anti-IgG (lanes 2, 6, 10, 14) antibody. Immunoprecipitated proteins were detected by immunoblotting with anti-Rta (lanes 1–8) or anti-TAF4 (lanes 9–16) antibody. Lanes 1, 5, 9 and 13 were loaded with 5% of the cell lysate.

Immunoprecipitation assay was conducted using the lysate from P3HR1 cells that had been treated with TPA and sodium butyrate for 24 hr to verify the interaction of Rta with TAF4 *in vivo*. The results reveal that Rta in the cell lysate was immunoprecipitated by anti-Rta antibody (Fig. 2, lane 4) and coimmunoprecipitated with TAF4 by anti-TAF4 antibody (Fig. 2, lane 3). TAF4 was also immunoprecipitated by anti-TAF4 antibody (Fig. 2, lane 12) and coimmunoprecipitated with Rta by anti-Rta antibody (Fig. 2, lane 11). Meanwhile, in a study that involved the lysate prepared from P3HR1 cells that had not been treated with TPA and sodium butyrate, immunoblotting revealed that Rta was not present in the lysate (Fig. 2, lane 5) and could not be immunoprecipitated by anti-Rta or anti-IgG antibody (Fig. 2, lanes 6, 8). Immunoblotting also revealed that TAF4 was present in the lysate (Fig. 2, lane 13) and was not immunoprecipitated by anti-IgG (Fig. 2, lane 14). Moreover, although anti-TAF4 antibody immunoprecipitated TAF4 in the lysate (Fig. 2, lane 16), did not coimmunoprecipitate Rta (Fig. 2, lane 7). Notably, a small amount of TAF4 was coimmunoprecipitated using anti-Rta antibody (Fig. 2, lane 15), perhaps because of the presence of a low level of Rta in the lysate due to spontaneous EBV lytic activation. Moreover, the specificity of anti-Rta antibody is significantly higher than that of anti-TAF4 antibody. Therefore, the amount of latent lysate loaded to the gel for the detection of Rta (Fig. 2. lanes 1–8) was substantially less than that for the detection of TAF4 (Fig. 2, lanes 9–16), explaining why an Rta band was undetected in the experiment (Fig. 2, lanes 5–8). Furthermore, Rta in the lysates that were prepared from cells that were treated with TPA and sodium butyrate was coimmunoprecipitated by anti-TAF4 antibody (Fig. 2, lane 3). Yet, we were unable to use the anti-TAF4 antibody to coimmunoprecipitate Rta in the lysates that were prepared from cells not infected by EBV (Fig. 2, lane 7), showing that anti-Rta antibody does not react to TAF4 nonspecifically.

### Colocalization of Rta and TAF4 in the nucleus

**Figure 3:**
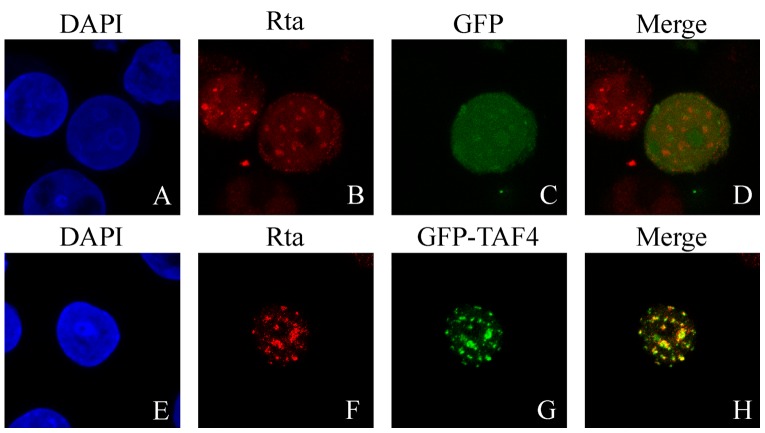
Indirect immunofluorescence analysis. P3HR1 cells were transfected with pEGFP-C1 (A–D) or pEGFP-TAF4 (E–H) and then treated with sodium butyrate for 24 hr. Cells were incubated with monoclonal anti-Rta antibody and observed under a confocal laser-scanning microscope. DAPI staining revealed the positions of nuclei (A and E). D and H are merged images.

A confocal microscopic work was performed to confirm the interaction between Rta and TAF4 in P3HR1 cells that were transfected with pEGFP-TAF4 and had been treated with sodium butyrate and TPA for 24 hr. The result showed that Rta colocalized with GFP-TAF4 as dots in the nucleus (Fig. 3H). Meanwhile, the cells transfected with an empty vector had GFP present diffusely in the entire cells and did not colocalize with Rta (Fig. 3C and 3D).

### Mapping the interaction domains in Rta and TAF4

**Figure 4:**
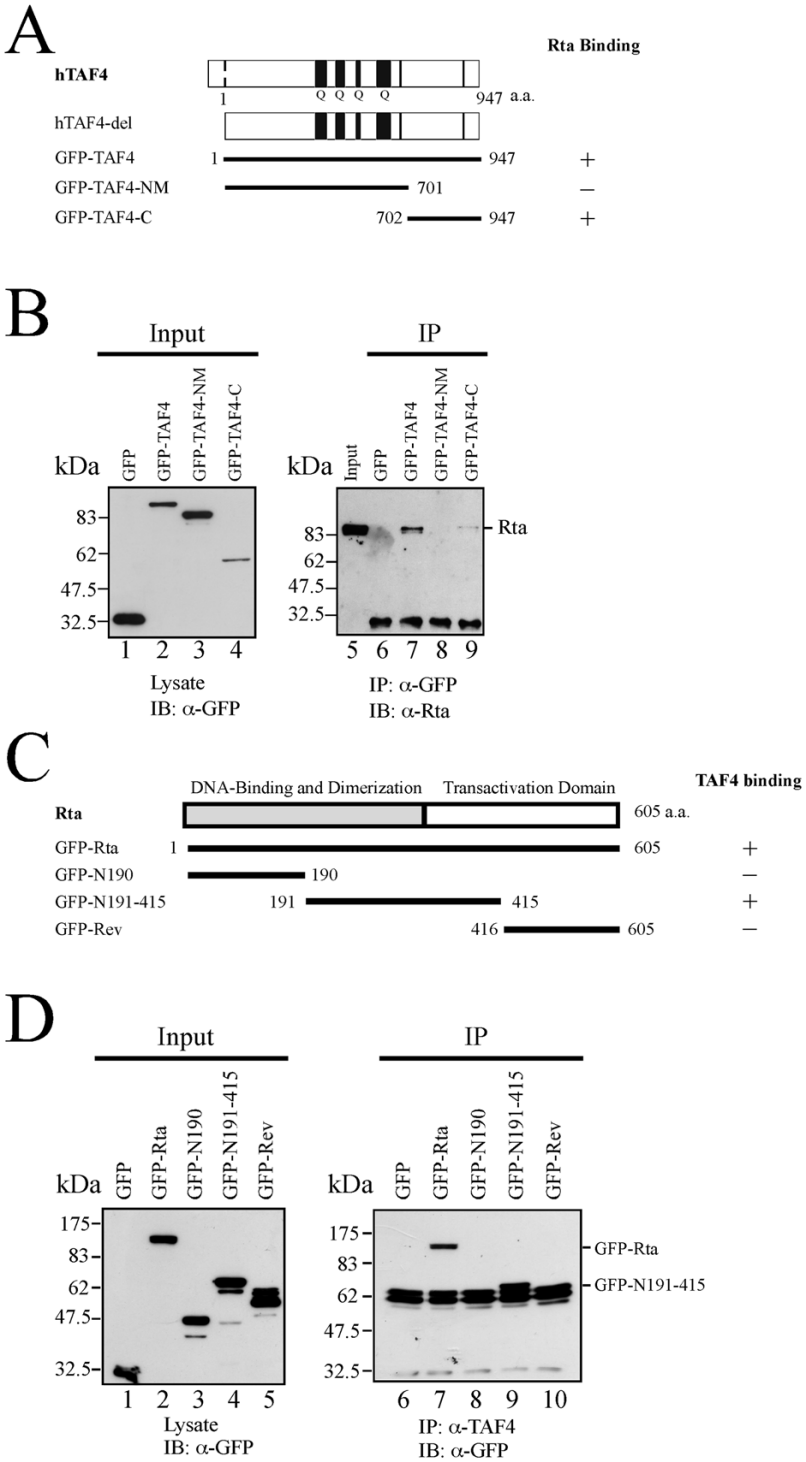
Mapping the interaction domains in TAF4 and Rta. (A) Plasmids that expressed deleted GFP-TAF4 were used to delineate the region in TAF4 that interacts with Rta. Numbers represent the positions of amino acids in TAF4. Q denotes the glutamine-rich regions. (B) 293T cells were cotransfected with pCMV-R and plasmids that expressed GFP fusion proteins including pEGFP-TAF4 (lanes 2, 7), pEGFP-TAF4-NM (lanes 3, 8), pEGFP-TAF4-C (lanes 4 and 9) or pEGFP-C1 (lanes 1, 6). Input lanes were loaded with 5% of the lysate (lanes 1–5). Proteins in the lysates were coimmunoprecipitated (IP) with anti-GFP antibody and analyzed by immunoblotting (IB) using anti-Rta antibody (lanes 6–9). (C) Deletion mutants of Rta were used to identify the region in Rta that interacts with TAF4. Numbers represent the positions of amino acids in Rta (D). Plasmids that expressed GFP-Rta (lanes 2, 7), GFP-N190 (lanes 3, 8), GFP-N191-415 (lanes 4, 9), GFP-Rev (lanes 5, 10) or GFP (lanes 1, 6) were transfected into 293T cells. The input lanes were loaded with 5% of the cell lysates and GFP-fusion proteins were detected using anti-GFP antibody (lanes 1–5). Proteins in the lysates were coimmunoprecipitated with anti-TAF4 antibody and analyzed by immunoblotting using anti-GFP antibody (lanes 6–10).

To delineate the regions in TAF4 that interact with Rta, 293T cells were cotransfected with pCMV-R and plasmids that encode GFP-TAF4, GFP-TAF4-NM, and GFP-TAF4-C (Fig. 4A). An empty vector, pEGFP-C1, that expressed GFP was used as a negative control. Immunoblotting using an anti-GFP antibody revealed the presence of GFP-TAF4 and GFP-TAF4-C in the lysate (Fig. 4B, lanes 2, 4). Immunoblotting using anti-Rta antibody also revealed that Rta was coimmunoprecipitated with GFP-TAF4 and GFP-TAF4-C by anti-GFP antibody (Fig. 4B, lanes 7, 9). A parallel experiment revealed that although GFP and GFP-TAF4-NM were present in the lysate (Fig. 4B, lanes 1, 3), Rta was not coimmunoprecipitated with these proteins by anti-GFP antibody (Fig. 4B, lanes 6, 8). These results suggested that the region between amino acids 702 and 947 in TAF4 interacts with Rta. Additionally, deletions were generated in GFP-Rta (Fig. 4C) to identify the regions in Rta that interacted with TAF4. These GFP-Rta fusion proteins were detected in the lysate by immunoblotting using the anti-GFP antibody after transfection (Fig. 4D, lanes 1–5). Immunoblot analysis revealed that only GFP-Rta and GFP-N191-415 were coimmunoprecipitated with TAF4 by anti-TAF4 antibody (Fig. 4D, lanes 7, 9), indicating that the region between amino acids 191 and 415 in Rta interacts with TAF4.

### Activation of BcLF1 transcription by interaction of Rta with TAF4

**Figure 5:**
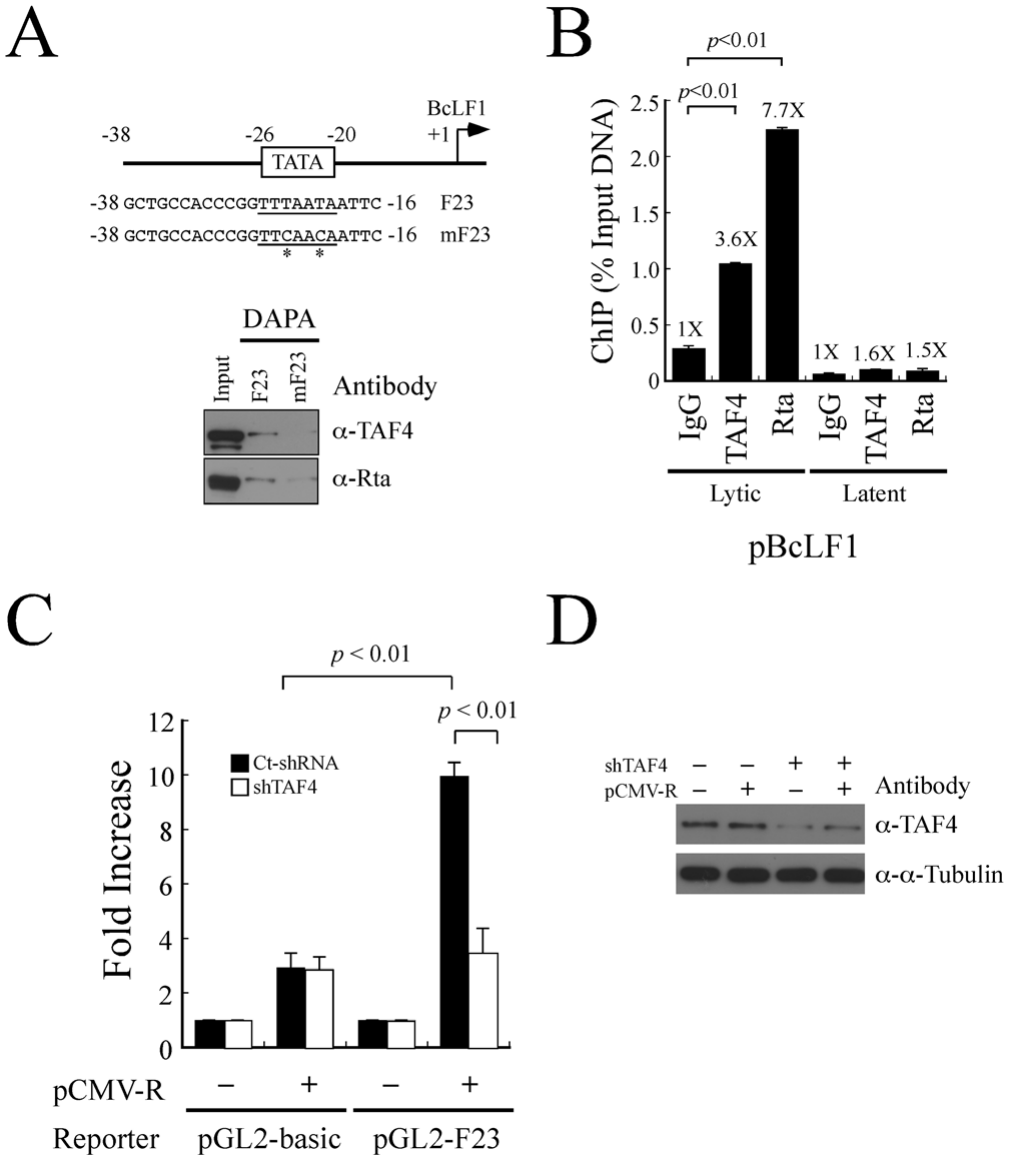
Involvement of TAF4 in the activation of the BcLF1 promoter by Rta. (A) Biotin-labeled double-stranded F23 probes were added to a lysate prepared from P3HR1 cells that had been treated with TPA and sodium butyrate for 52 hr. Mutant probes mF23 that contains mutated sequences (asterisk) was used as negative controls. Proteins bound to the probes were captured by the streptavidin magnetic beads and detected by immunoblotting analysis using anti-TAF4 and anti-Rta antibodies. Input lanes were loaded with 5% of the cell lysate. DAPA: DNA affinity precipitation assay. (B) P3HR1 cells were treated with TPA and sodium butyrate for 52 hr. Formaldehyde-fixed DNA-protein complex was immunoprecipitated using anti-TAF4 or anti-Rta antibody. The reaction with added anti-IgG antibody was used as a negative control. The binding of TAF4 and Rta to the BcLF1 promoter was investigated by qPCR. Error bar represents standard error. (C) 293T cells were cotransfected with pCMV-R and pGL2-F23 or a control vector pGL2-basic in the presence of control shRNA (Ct-shRNA) (filled column) or TAF4 shRNA (shTAF4) (empty column). Luciferase activity was detected at 48 hr after transfection. Each transfection experiment was performed three times, and each sample in the experiment was prepared in duplicate. (D) The effect of TAF4 shRNAs was examined by immunoblotting with anti-TAF4 and anti-α-tubulin antibodies. The *p* value from each experiment was analyzed statistically with the Student's t- test method.

Rta is known to activate a number of EBV late genes, including BcLF1 [17]. Previous studies have found that the BcLF1 gene is transcribed from a promoter that is 23 bp long, which includes a TATA box [43], [47]. Hence, this study investigates whether Rta interacts with TAF4 to activate the transcription of BcLF1. Accordingly, DAPA was conducted using a biotin-labeled F23 DNA probe that contained the 23-bp sequence, from -38 to -16, in the BcLF1 promoter (Fig. 5A). The probe was added to a lysate that was prepared from P3HR1 cells that had been treated with sodium butyrate and TPA. Proteins that were bound to the probe were captured using streptavidin magnetic beads. Immunoblot analysis revealed the binding of TAF4 and Rta to the F23 probe. However, TAF4 and Rta did not bind to a mutant probe, mF23, which contains a mutated TATA sequence (Fig. 5A), indicating the binding of Rta and TAF4 to the TATA sequence in the BcLF1 promoter. Furthermore, ChIP analysis was performed to confirm the binding of TAF4 and Rta to the TATA box in the BcLF1 promoter *in vivo*. After P3HR1 cells were treated with sodium butyrate and TPA for 52 hr to induce the expression of EBV late genes, proteins and DNA in the cells were crosslinked with formaldehyde. qPCR with primers that were specific for the BcLF1 promoter sequence was subsequently performed. Both TAF4 and Rta were found to be bound to the BcLF1 promoter (Fig. 5B). The amounts of the BcLF1 promoter captured by anti-TAF4 and anti-Rta antibodies was 3.6 and 7.7 times, respectively, the amounts that were immunoprecipitated by anti-IgG antibody (Fig. 5B). Furthermore, only a background level of BcLF1 promoter DNA was immunoprecipitated by anti-IgG, anti-TAF4, and anti-Rta antibodies when P3HR1 cells were not treated with sodium butyrate and TPA (Fig. 5B). These results indicate the binding of Rta and TAF4 to the BcLF1 promoter *in vivo*. Transient transfection assay was also carried out in 293T cells that were transfected with pGL2-F23, which contains the region from nucleotides -38 to -16 in the BcLF1 promoter to investigate whether TAF4 is involved in Rta-mediated BcLF1 transcription. After transfecting 293T cells with pCMV-R, the activity of the BcLF1 promoter increased by tenfold (Fig. 5C). Meanwhile, transfecting two plasmids that express the shRNAs of TAF4 decreased the expression of TAF4 in the cells (Fig. 5D) and reduced by 70% the activity of the BcLF1 promoter that was activated by Rta (Fig. 5C). The expression of shRNA did not influence the luciferase activity of pGL2-basic that was nonspecifically activated by Rta (Fig. 5C) [13], [48]. These results suggested that the activation of BcLF1 transcription by Rta requires TAF4.

### Involvement of the Sp1-TAF4-Rta complex to Sp1-binding sites in TATA-less promoters of EBV

**Figure 6:**
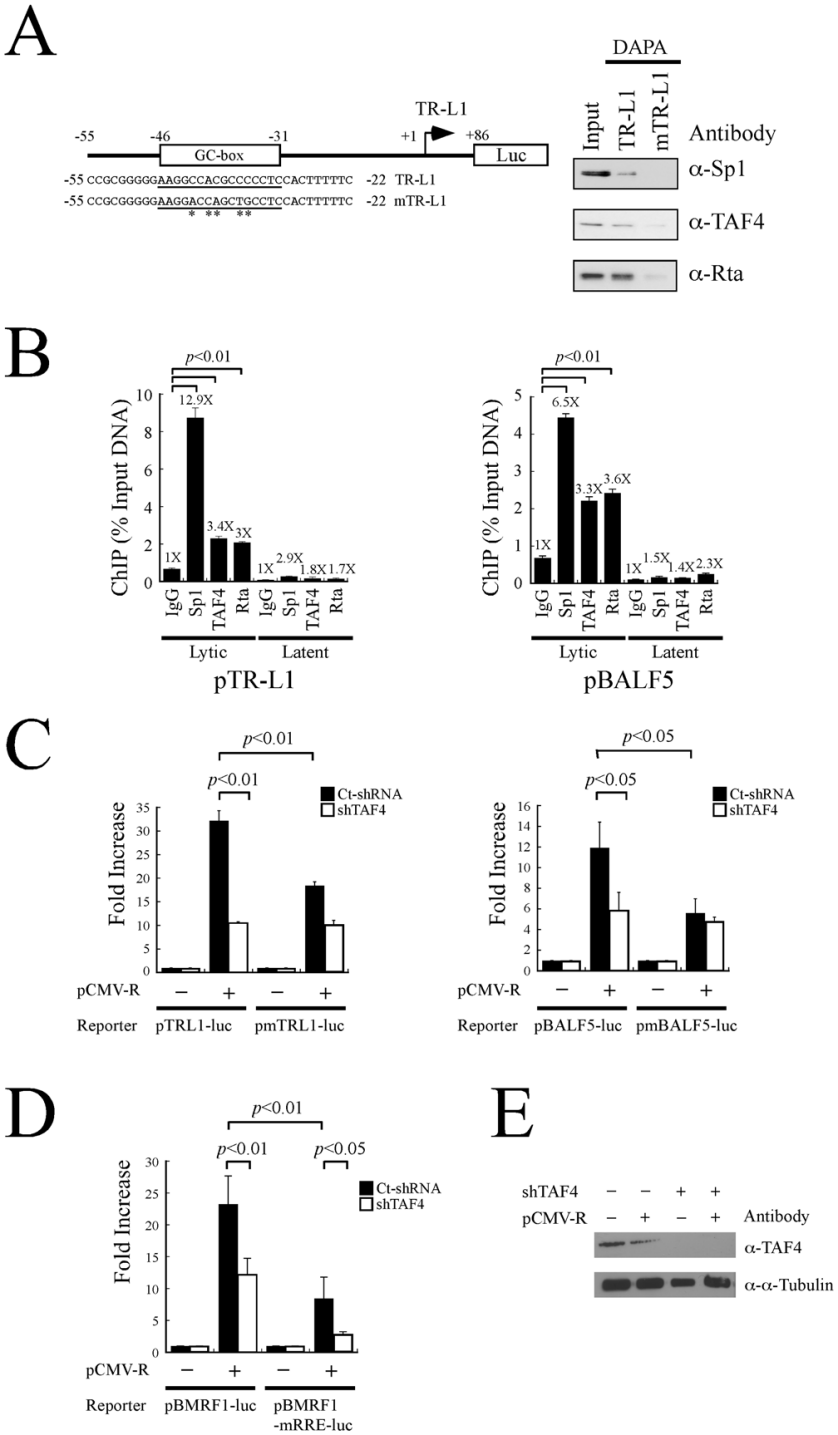
Influence of TAF4 on the activation of EBV TATA-less promoters by Rta. (A) The TR-L1 reporter plasmid and sequences of biotin-labeled probes, TR-L1 and mTR-L1. The probes were added to a lysate prepared from P3HR1 cells that had been treated with TPA and sodium butyrate. Proteins bound to the probes were captured by streptavidin magnetic beads and detected by immunoblotting analysis using anti-Sp1, anti-TAF4 and anti-Rta antibodies. Input lanes were loaded with 5% of the cell lysate. DAPA: DNA-affinity precipitation assay. (B) P3HR1 cells that had been treated with TPA and sodium butyrate for 48 hr (lytic) or treated with DMSO (latent) were fixed with formaldehyde and the DNA-protein complexes were immunoprecipitated using anti-Sp1, anti-TAF4 and anti-Rta antibodies. The binding of Sp1, TAF4 and Rta to the TR-L1 and BALF5 promoters was investigated by qPCR. Error bar represents standard error. (C) 293T cells were cotransfected with pCMV-R and reporter plasmids, including pTRL1-luc and pBALF5-luc in the presence of control shRNA (Ct-shRNA) (filled column) or TAF4 shRNA (empty column). Luciferase activities were monitored at 48 hr after transfection. Each transfection experiment was performed at lease three times, and each sample in the experiment was prepared in duplicate. (D) A similar experiment in (C) was performed using pBMRF1-luc and pBMRF1-mRRE-luc. (E) The effect of TAF4 shRNAs on the expression of TAF4 was examined by immunoblotting using anti-TAF4 and anti-α-tubulin antibodies. The *p* value from each experiment was analyzed statistically with the Student's t- test method. Luc: luciferase gene.

This study investigates whether the transcriptional activation of the TR-L1 promoter of the EBV BNLF1 gene, which lacks a TATA box, by Rta [49] involves TAF4. A biotin-labeled TR-L1 DNA probe, which contains the region from -32 to -47 in the TR-L1 promoter (Fig. 6A), was added to a lysate that was prepared from P3HR1 cells that had been treated with sodium butyrate and TPA. Proteins that bound to the probe were then captured using streptavidin magnetic beads. Immunoblot analysis revealed the binding of Sp1, TAF4 and Rta to the probe (Fig. 6A). These proteins did not bind to a mutant probe, mTR-L1, whose sequence is identical to that of TR-L1, except for a mutated Sp1-binding sequence (Fig. 6A). After the binding of Sp1, TAF4, and Rta to the Sp1-binding site in the promoter *in vitro* was demonstrated, the binding of the complex to the Sp1 site *in vivo* was studied by ChIP analysis. Accordingly, P3HR1 cells were treated with sodium butyrate and TPA to induce EBV lytic activation and cells that were treated with DMSO were used as a control. Following the immunoprecipitation with anti-Sp1, anti-TAF4 and anti-Rta antibodies, qPCR analysis using primers that amplified the Sp1-binding sequences in the TR-L1 promoter revealed that these three antibodies immunoprecipitated the DNA fragments that contained the TR-L1 promoter. The amounts of promoter sequences that were captured by anti-Sp1, anti-TAF4 and anti-Rta antibodies were 12.9, 3.4, and three times higher than that immunoprecipitated by anti-IgG antibody (Fig. 6B). Additionally, when cells were treated with DMSO rather than TPA and sodium butyrate, only the background amount of TR-L1 promoter was amplified by qPCR (Fig. 6B). A similar result was obtained using the BALF5 promoter of EBV, which also lacks a TATA sequence, in P3HR1 cells that had been treated with sodium butyrate and TPA for 48 hr to induce the expression of Rta. qPCR analysis using primers that were specific for the Sp1-binding sites in the BALF5 promoter demonstrated that these three antibodies immunoprecipitated the BALF5 promoter sequences in amounts that were 6.5, 3.3, and 3.6 times that obtained using the anti-IgG antibody, respectively (Fig. 6B). Meanwhile, background levels of the DNA fragment were captured by these antibodies when the cells were untreated with TPA and sodium butyrate (Fig. 6B). shRNA that inhibits the expression of TAF4, was also used to demonstrate the participation of Rta and TAF4 in transcriptional activation of these two TATA-less promoters. Immunoblotting verified that TAF4 expression declined to an undetectable level after 293T cells were transfected with two plasmids that expressed shRNAs of TAF4 (Fig. 6E). Thereafter, 293T cells were cotransfected with pTRL1-luc and pCMV-R in the presence of TAF4 shRNA or control shRNA. The results showed that introducing TAF4 shRNA reduced the transcriptional activity of the TR-L1 promoter that was activated by Rta from 32-fold to tenfold (Fig. 6C). This study also used pmTRL1-luc, which contains a *luc* gene transcribed from a mutant TR-L1 promoter that lacks the Sp1 site. Despite of the mutation, Rta activated the promoter 18-fold; the expression of TAF4 shRNAs further reduced the promoter activity to tenfold (Fig. 6C), revealing the involvement of TAF4 in Rta-mediated TRL1 transcription. A parallel experiment demonstrated that overexpressing Rta increased the luciferase activity of pBALF5-luc by a factor of 12 (Fig. 6C), but only by a factor of six after shRNA had been expressed (Fig. 6C). When the reporter plasmid that contained a mutated Sp1 binding sites, pmBALF5-luc, was utilized, Rta increased the promoter activity by a factor of 5.6 (Fig. 6C). Silencing of TAF4 expression did not influence the activity of the mutant BALF5 promoter (Fig. 6C). These results suggest that TAF4 is important to the transcriptional activation of the TR-L1 and BALF5 promoters by Rta.

Rta is known to bind directly to RRE to affect transcription [4], [6]–[11]. A transient transfection study was performed using the promoter pBMRF1-luc that contains an RRE [18] to investigate whether TAF4 also participates in RRE-dependent transcription. Accordingly, luciferase activity was monitored by cotransfecting 293T cells with pBMRF1-luc and pCMV-R. As expected, Rta activated the promoter activity of pBMRF1 23-fold (Fig. 6D). The increase in promoter activity that was exhibited by pBMRF1-luc was decreased to 12-fold after the expression of TAF4 shRNA (Fig. 6D). When 293T cells were cotransfected with a reporter plasmid that contained a mutated RRE, pBMRF1-mRRE, and pCMV-R, the luciferase activity of pBMRF1-mRRE was eight times that of the negative control (Fig. 6D). Knockdown of TAF4 expression by shRNA reduced the increase in activity of the promoter to threefold (Fig. 6D), showing that Rta-TAF4 complex does not need RRE to activate transcription.

### Function of TAF4 in the transcriptional activation of human androgen receptor (hAR) by Rta

**Figure 7:**
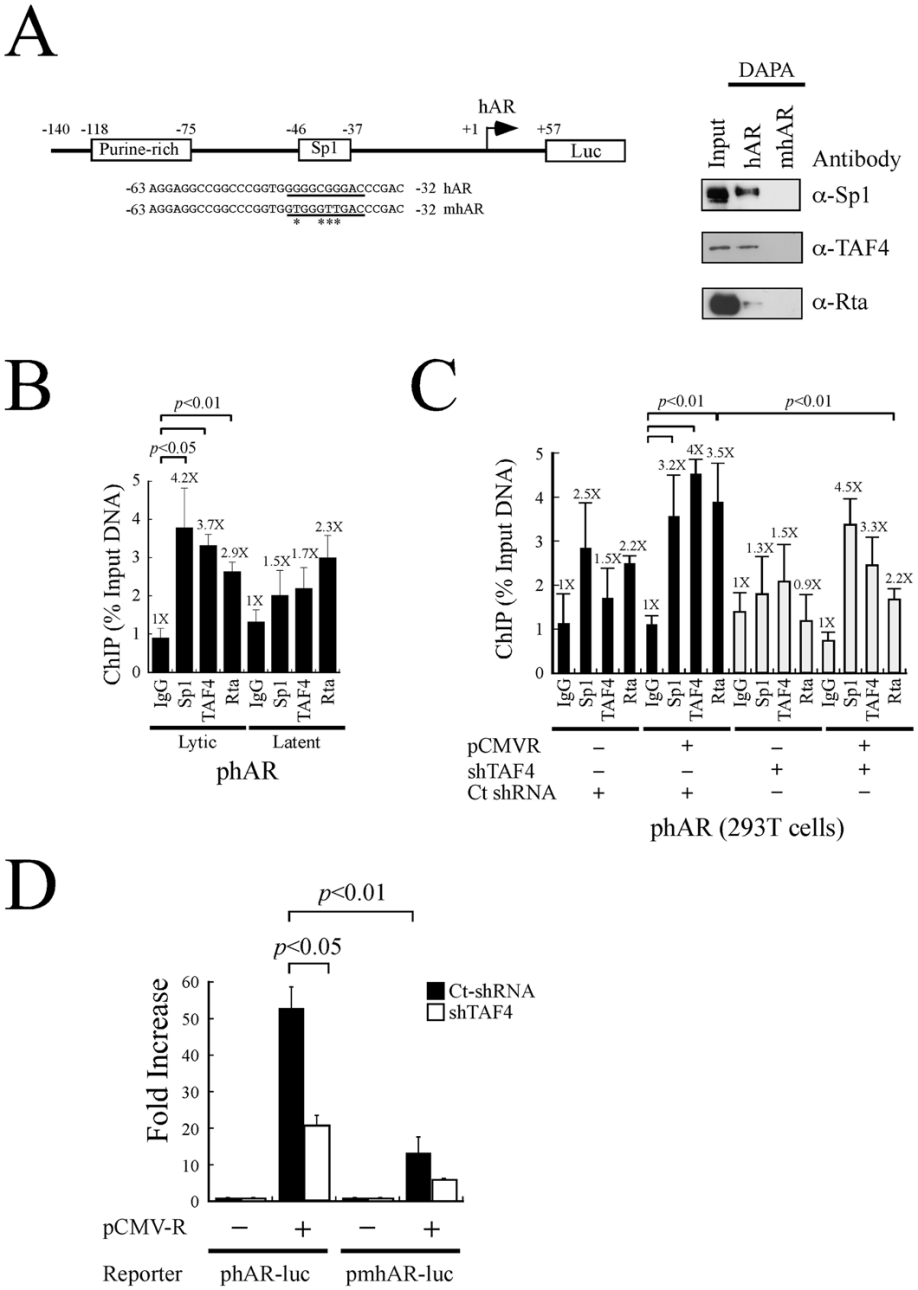
Role of TAF4 in the transcription of human androgen receptor by Rta. (A) An hAR reporter plasmid and sequences of biotin-labeled probes, hAR and mhAR. The probes were added to a lysate prepared from P3HR1 cells that had been treated with TPA and sodium butyrate. Proteins bound to the probes were captured by streptavidin magnetic beads and detected by immunoblot analysis using anti-Sp1, anti-TAF4 and anti-Rta antibodies. Input lanes were loaded with 5% of the cell lysate. DAPA: DNA-affinity precipitation assay. (B) P3HR1 cells that had been treated with TPA and sodium butyrate for 48 hr (lytic) or treated with DMSO (latent) were fixed with formaldehyde and the DNA-protein complexes were immunoprecipitated with anti-Sp1, anti-TAF4 and anti-Rta antibodies. The binding of Sp1, TAF4 and Rta to the hAR promoter was investigated by qPCR. (C) 293T cells were cotransfected with pCMV-R and TAF4 shRNA (empty column) or control shRNA (filled column) for 48 hr. CHIP assay was subsequently performed. The reaction with added anti-IgG antibody was used as a negative control. Error bar represents standard error. (D) 293T cells were cotransfected with pCMV-R and a reporter plasmid including phAR-luc or pmhAR-luc, in the presence of control shRNA (Ct-shRNA) (filled column) or TAF4 shRNA (empty column). Luciferase activities were monitored at 48 hr after transfection. Each transfection experiment was performed at lease three times, and each sample in the experiment was prepared in duplicate. The *p* value from each experiment was analyzed statistically with the Student's t- test method. Luc: luciferase gene.

In this study, the promoter from human androgen receptor gene, which lacks a TATA box [44], was used to determine whether TAF4 is involved in Rta-activated transcription. The DAPA results indicated that Sp1, TAF4 and Rta were bound to the probe, but did not bind to the mutant probe that contained a mutated Sp1 site (Fig. 7A). ChIP assay revealed that when P3HR1 cells were treated with TPA and sodium butyrate, the amounts of Sp1, TAF4, and Rta bound to the hAR promoter were 4.2-, 3.7-, and 2.9-fold higher than those captured by anti-IgG antibody (Fig. 7B). When cells were not treated with TPA and sodium butyrate, these antibodies captured only background amount of the promoter DNA (Fig. 7B). This study introduces TAF4 shRNA into 293T cells to examine how TAF4 influences the transcription of the androgen receptor gene that is activated by Rta. Accordingly, ChIP assay was performed using 293T cells that were cotransfected with pCMV-R and TAF4 shRNA or control shRNA for 48 hr. qPCR analysis using primers to amplify the hAR promoter indicated that overexpressing Rta increased the amounts of Sp1, TAF4, and Rta bound to the promoter by factors of 3.2, 4, and 3.5, respectively (Fig. 7C). However, introducing TAF4 shRNA reduced the levels of TAF4 and Rta that bound to the promoter from 4-fold to 3.3-fold and from 3.5-fold to 2.2-fold, respectively (Fig. 7C), but did not influence the binding of Sp1 to the promoter (Fig. 7C). A transient transfection experiment was conducted using a reporter plasmid phAR-luc. Overexpressing Rta increased the luciferase activity of phAR-luc 53-fold (Fig. 7D), but only 21-fold following the expression of the TAF4 shRNA (Fig. 7D), The increase in Rta-activated activity of a mutant hAR promoter in pmhAR-luc, which contains a mutated Sp1 site, declined from 13-fold to sixfold when TAF4 shRNA was cotransfected (Fig. 7D).

## Discussion

Rta is a transcription factor that is expressed by EBV in the immediate-early stage of the lytic cycle, to activate the transcription of EBV lytic genes by binding to RRE in promoters [11] or by forming a complex with Sp1 or Zta by interacting with MCAF1 [13], [18]. Rta also activates TATA-less and GC-rich promoters of EBV, including BNLF1 and BALF5 [10], [49]. Furthermore, Rta interacts with basal transcription factors, TBP and TFIIB, to activate transcription [38]. This study demonstrates that Rta interacts with a core component of TFIID, TAF4, to activate both TATA and TATA-less transcription, revealing how Rta interacts with the eukaryotic transcription machinery to activate transcription.

Several lines of evidence indicate that TFIID binding to promoters is a rate-limiting step in transcription [22], [23], [50] and that transcription factors such as Sp1 or CREB recruits and stabilizes TFIID at the TATA sequence [28], [51]–[53]. This study demonstrates that Rta interacts with TAF4 by GST-pulldown assay (Fig. 1), coimmunoprecipitation (Fig. 2), and confocal microscopy (Fig. 3H). The result shows that a small amount of TAF4 that was coimmunoprecipitated by anti-Rta antibody (Fig. 2, lane 15). Because P3HR1 is known to express Rta in a cell population that is untreated with TPA and sodium butyrate due to spontaneous EBV lytic activation, coimmunoprecipitation of TAF4 by anti-Rta antibody is likely attributed to the leaky expression of Rta. Additionally, the interaction involves the C-terminal domain in TAF4 and the region between amino acids 191 and 415 in Rta (Fig. 4). Previous studies have shown that the C-terminal region (623–921 a.a.) of TAF4, which is conserved from yeast to humans, stabilizes and nucleates the holo-TFIID complex [27], [54], suggesting that Rta promotes and stabilizes the assembly of the TFIID complex by interacting with the C-terminal region in TAF4.

This study demonstrates that Rta cooperates with TAF4 to activate promoters that contain a TATA sequence. A previous study demonstrated that the activation by Rta of the transcription of BcLF1 through a 23-bp region, from nucleotide -38 to -16, which contains a TATA box, is sufficient for the promoter activity [43], [47]. Hence, Rta probably interacts with basal transcription factors that bind to the TATA box, influencing the BcLF1 transcription. The DAPA and ChIP assay herein reveal that Rta and TAF4 form a complex on the BcLF1 promoter upon lytic induction (Fig. 5A and 5B). Transient transfection analysis also shows that introducing TAF4 shRNA reduces the ability of Rta to activate the transcription of BcLF1 by 70% (Fig. 5C), indicating that Rta activates the transcription of BcLF1 by interacting with TAF4. However, this study cannot exclude the possible involvement of other EBV-encoded protein, such as BcRF1, which is a TBP-like protein that forms a complex on TATA motifs in the activation of late genes [55].

This study shows that the interaction of Rta and TAF4 activates the EBV promoters that lack a TATA sequence. Earlier studies have established that Rta transcriptionally activates two EBV promoters without a TATA sequence, the TR-L1 promoter of BNLF1 and the promoter of BALF5, after lytic activation [49], [56]. Rta is demonstrated to interact with TAF4 and Sp1 and form a complex on the promoters of TR-L1 and BALF5 after EBV lytic induction (Fig. 6B). Additionally, TAF4 shRNA substantially reduces the ability of Rta to activate these two promoters (Fig. 6C), revealing that the activation of EBV TATA-less promoters by Rta depends on the formation of the Sp1-TAF4-Rta complex and subsequent binding of this complex to the Sp1 site. Rta activates the TR-L1 promoter at a reduced level after the Sp1 site in the promoter is mutated (Fig. 6C), which may be attributed to the fact that the TR-L1 promoter contains high GC-rich sequences, such as the region from -3 to -14 [37], [57], where Sp1 may also bind, allowing Rta to activate transcription through this sequence.

This study shows that Rta considerably activates the hAR promoter, which has no TATA sequence (Fig. 7D). Mutation of the Sp1 sequence in the promoter reduces the luciferase activity that was activated by Rta (Fig. 7D), implying that Rta activates the hAR promoter via its Sp1-binding sequence. The DAPA and ChIP analysis reveal that Rta, TAF4, and Sp1 form a complex on the hAR promoter after lytic induction (Fig. 7A, 7B). Additionally, ChIP analysis revealed that the knockdown of TAF4 expression reduces the amount of Rta but not the amount of Sp1 on the promoter (Fig. 7C), indicating that Rta is recruited by TAF4 and then interacts with the Sp1-DNA complex. The fact that introducing TAF4 shRNAs reduces the promoter activities and the amount of Rta-TAF4 that is bound to the promoter (Fig. 7C, 7D) also indicates that TAF4 is important to the activation of the hAR promoter by Rta. This study finds that Rta is capable of activating a mutant hAR promoter that lacks an Sp1-binding site although to a reduced extent (Fig. 7D), suggesting that Rta may affect hAR transcription through factors other than Sp1 and TAF4.

Rta is known to activate many EBV lytic promoters by binding to RRE [4], [6]–[11]. This study finds that after changing the RRE sequence in the BMRF1 promoter, the mutation does not completely eliminate the ability of Rta to activate the promoter. The increase in promoter activity was reduced from 23-fold to eightfold (Fig. 6D). Meanwhile, the knockdown of the expression of TAF4 reduces the ability of Rta to activate the transcription from eightfold to a threefold increase (Fig. 6D), showing that wild-type pBMRF1 and mutant pBMRF1 have similar levels when TAF4 shRNA was cotransfected. This result implies that Rta-TAF4 complex does not need RRE to activate transcription. Rta and Zta are two transcription factors that critically influence the lytic development of EBV. The results of this study and other investigations [58]–[61] indicate that both transcription factors interact with TFIID, promoting transcription. However, the activation by Zta seems to be limited to a subset of EBV lytic promoters that contain ZRE and Zta does not activate a promoter without a TATA sequence [18]. However, Rta uses a different strategy, interacting with many transcription factors, including TAF4, Sp1, Zta, MCAF1, and RanBPM, to activate the transcription of both viral and cellular [13], [18], [62], verifying that Rta activates a broad spectrum of EBV genes [63]. These findings indicate that the two EBV immediate-early transcription factors, although both interact with basal transcription factors, act differently but synergistically to promote the lytic development of EBV.
